# Need for Identification Cards for Patients With Teeth Treated With Regenerative Endodontic Procedure: A Report of Two Cases

**DOI:** 10.7759/cureus.105216

**Published:** 2026-03-14

**Authors:** Arieh Y Kaufman, Nooshin Noghreian, Margarita Yoshpe

**Affiliations:** 1 Endodontology, The Maurice and Gabriela Goldschleger School of Dental Medicine, Gray Faculty of Medicine and Health Sciences, Tel Aviv University (Retired), Tel Aviv, ISR; 2 Endodontics, Loma Linda University School of Medicine, Loma Linda, USA; 3 Dentistry, Hadasah Dental School, Hebrew University, Jerusalem, ISR

**Keywords:** calcification, iatrogenic damage, identification card, perforation, regenerative endodontic procedure

## Abstract

Regenerative endodontic procedures (REPs) were initially designed to treat young, immature teeth. Organisations commonly endorse REP as a valid treatment for non-vital immature teeth. Despite its effectiveness, general dentists often lack exposure to REPs during their training, leading to limited awareness of the procedure. This knowledge gap amongst general dentists and specialists can result in misdiagnosis and inappropriate treatment of REP-treated teeth years after the initial procedure. Therefore, we present two cases in which the misdiagnosis of long-term successful REP-treated teeth led to improper treatment, causing iatrogenic damage to long-standing successful REP-treated teeth. To mitigate such risks, we propose introducing a treatment identification card that briefly describes the REP treatment and recommendations for further treatments.

## Introduction

Regenerative endodontic procedures (REPs) were initially designed to treat young immature teeth [1]. Professional organizations such as the American Association of Endodontists (AAE) [1], the American Academy of Pediatric Dentistry [2], and the European Academy of Paediatric Dentistry (EAPD) endorse REP as a valid treatment for non-vital immature teeth, although the EAPD recommends its use in limited cases [3]. More than 626 publications have documented favorable outcomes for REP. The success rates of REP for eliminating periapical lesions range from 90% to 100% [4,5]. Additional advantages include root canal lengthening and widening [6,7], treatment of immature non-vital posterior teeth [8], arresting external inflammatory root resorption, and external replacement root resorption (ERRR) [9]. REP-treated teeth have also been shown to respond well to orthodontic forces when applied in a controlled manner [10]. Despite its effectiveness, REP is primarily practiced by endodontists and pediatric dentists, and general dentists often lack exposure to REP during their training, leading to limited awareness of the procedure [11]. This knowledge gap among general dentists and specialists can result in misdiagnosis and inappropriate treatment of REP-treated teeth years after the initial procedure. Radiopaque materials in the coronal third of the root canal or calcifications within the canal may be misinterpreted as pathology, leading to unnecessary retreatment with unfavorable outcomes. To address these issues, this paper presents two cases in which REP-treated teeth underwent unnecessary retreatment due to the clinician's unfamiliarity with REP. Given these challenges, the present study aims to introduce a simple identification card (ID RET card) for patients who have undergone REP, providing clinicians with an immediate and reliable reference that may help prevent diagnostic errors and support appropriate treatment planning, tooth number, and recommendations for future care, for each REP-treated tooth.

## Case presentation

Case 1

**Figure 1:**
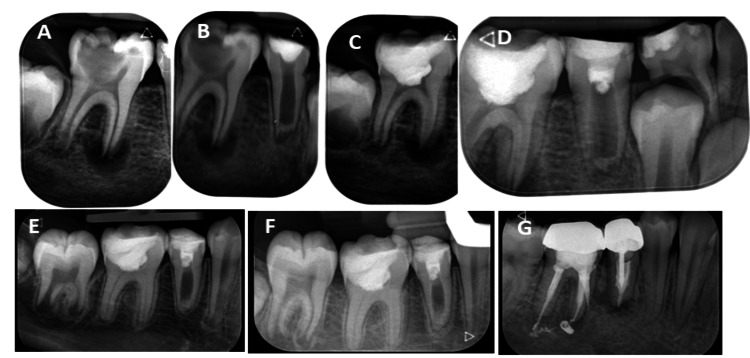
Case 1 - radiographic images (A) Radiograph of tooth #30 upon first arrival at the office. (B) Radiograph of tooth #29 upon first arrival at the office. (C) Radiograph of tooth #30 at the end of vital pulp therapy. (D) Radiograph of tooth #29 at the end of regenerative endodontic treatment. (E) Follow-up radiograph at 28 months. A traceable periodontal ligament along both roots of tooth #30 and more dentin in the apical area of the canal of tooth #29 were noted. (F) Follow-up radiograph at 46 months. A traceable periodontal ligament along both roots of tooth #30 and more calcified tissue in the pulp of tooth #29 were noted. (G) Follow-up radiograph at 100 months. Teeth #29 and #30 were treated with root canals and crowns.

A 9-year-old girl presented with a periapical lesion associated with deep caries in tooth #30. The initial clinical examination included a comprehensive assessment of the tooth, consisting of extra‑ and intra‑oral inspection, percussion and palpation tests, thermal and electric sensibility testing, and evaluation of mobility and periodontal status. These findings were used to establish the baseline condition and guide the diagnostic process. Initial examination revealed necrotic pulp and asymptomatic apical periodontitis (Fig. 1a). Clinically, the tooth was asymptomatic, with no tenderness to percussion or palpation. Radiographically, the tooth exhibited open apices in both the mesial and distal roots. The periapical lesion was associated with the mesial root. Dental history revealed that an endodontic treatment was initiated in tooth #29 approximately 1 year prior but was never completed (Fig. 1b). No additional information regarding the initial treatment was available. Sensibility tests were negative, with no tenderness to percussion.

Radiographic evaluation demonstrated open root apices, with a large radiolucent area associated with the mesial root and incomplete development of the distal root. Clinical examination revealed normal periodontal probing depths and mobility tests. A diagnosis of necrotic pulp with asymptomatic apical periodontitis was established. After informed consent was obtained from the patient’s parents, root canal treatment was initially planned. After local anesthesia (lidocaine 2% with 1:100,000 epinephrine; Safco, Buffalo Grove, USA) was administered, treatment was initiated using a dental operating microscope (Labomed Prima, Los Angeles, USA). The injection technique used for local anesthesia was an inferior alveolar nerve block. Caries was removed, and the pulp chamber was accessed. During treatment, vital pulp tissue was unexpectedly observed, prompting a modification of the treatment plan to vital pulp therapy. Hemostasis was achieved with gentle pressure using a cotton pellet soaked in 3% NaOCl (sodium hypochlorite).

Mineral trioxide aggregate (MTA; Angelus Odontologia, Londrina, Brazil) was placed, and the tooth was sealed with a glass ionomer (Fuji; GC Corporation, Tokyo, Japan) and a packable composite resin (Filtek P60; 3M Centre, St. Paul, USA) (Fig. 1c). Clinical examination of tooth #29 revealed negative sensibility, percussion, and palpation test results. Probing depth and mobility test results were within normal limits. Radiographic examination demonstrated a wide root canal space closed at its apex and surrounded by a periapical radiolucent area measuring approximately 2 × 6 mm. Diagnosis of a previously initiated endodontic therapy with asymptomatic apical periodontitis was made. After discussing treatment options with the patient’s parents, regenerative endodontic treatment (RET) was recommended. The objective was to promote disinfection, periapical pathology healing, root wall thickening, and continued root development. After the parents provided consent, RET was performed according to the AAE protocol [12].

The canal was irrigated with 3% NaOCl, dried, and medicated with a triple antibiotic paste comprising equal parts (250 mg each) of metronidazole (Sanofi, Paris, France), cefuroxime axetil (Zinnat; GSK, London, UK), and ciprofloxacin (Dexcel, Or Akiva, Israel), mixed with saline to a creamy consistency. The access cavity was temporarily sealed with interim restorative material** **(Dentsply Sirona, Bensheim, Germany) for 3 weeks. RET was completed at the second appointment using platelet-rich fibrin (PRF) to create a scaffold as previously described by Yoshpe et al. [6,8] (Fig. 1d). A collagen pellet was placed over the PRF, followed by placement of MTA as the coronal barrier. Follow-up examinations at 28 and 46 months showed resolution of periapical lesions, intact periodontal ligament surrounding the roots of tooth #30, further apical development of the distal root, and progressive calcification in the root canal of tooth #29 (Fig. 1e, 1f). The patient did not attend any further recall appointments until March 2025 (100-month recall). At that time, radiographic examination revealed that both teeth had undergone unnecessary, poor-quality root canal treatment due to a misdiagnosis (Fig. 1g).

Further inquiry revealed successful outcomes for both teeth until January 2023. According to the patient’s report, she sought dental care because of a fractured restoration. The dentist misdiagnosed the REP treatment as an internal resorption in tooth #29, initiated and completed root canal therapy, and placed a post and performed crown restoration in both teeth.

This case highlights the importance of long-term follow-up and a clear explanation to the patient and her parents regarding the uniqueness** **of REP treatment to prevent subsequent unnecessary and potentially harmful dental interventions and retreatments.

Case 2

**Figure 2:**
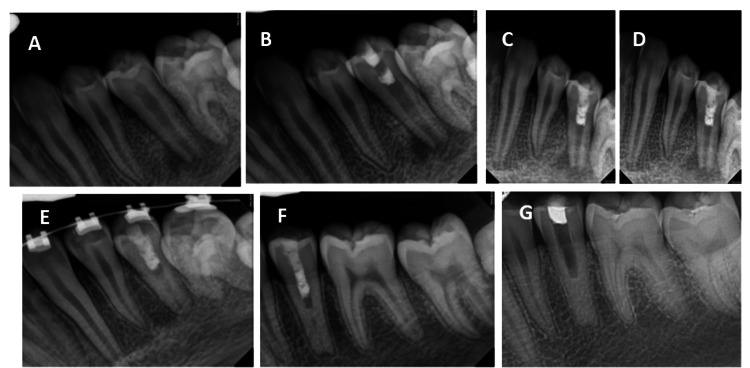
Case 2 - radiographic images (A) Preoperative radiograph (October 2012) showing tooth #20 with an open apex and periapical radiolucency. (B) Radiograph after the initial regenerative endodontic procedure showing placement of mineral trioxide aggregate and CollaCote barrier (OsseoDent, Miami, USA). (C) Follow-up radiograph at 4 months (February 2013) showing persistent periapical pathology prompting retreatment. (D) Radiograph obtained on the same day showing completed repeat regenerative endodontic procedure using mineral trioxide aggregate and CollaCote barrier. (E) Recall visit radiograph at 10 months showing thickening of dentinal walls and an increase in root length. (F) Recall visit radiograph at 14 months showing increased calcification of the apical half of the canal space and normal root length. (G) Radiograph showing an empty canal space with temporary restoration.

A 12-year-old boy was referred by a pediatric dentist to an endodontic practice for an evaluation of the lower left area. He presented with pain and discomfort localized to tooth #20, especially during brushing. Clinical examination revealed tenderness to percussion and grinding of tooth #20, which was non-responsive to cold and electric pulp testing. Periodontal probing was 4 mm on mesiobuccal, and a remnant of a sinus tract on the buccal aspect of tooth #20 was observed. Radiographic evaluation revealed an immature apex and signs consistent with apical periodontitis (Fig. 2a). The diagnosis was non-vital pulp and symptomatic apical periodontitis in tooth #20. A pulpal regenerative procedure was initiated using **Scandonest** 3% plain (Septodont, Ontario, Canada) for anesthesia of tooth #20. The local anesthetic injection technique used for tooth #20 was the inferior alveolar nerve block. The canal was accessed and gently disinfected with 1.25% NaOCl, followed by a final rinse with 17% ethylenediaminetetraacetic acid (EDTA).

Bleeding was initiated to create a scaffold, and a collagen matrix (CollaCote; OsseoDent, Miami, USA) was placed on top of the clot, which filled the entire canal. MTA (ProRoot MTA; Dentsply Sirona, Charlotte, USA) was placed coronally. A moist cotton pellet was placed on top of the MTA to ensure the setting of the material. Cavit G (3M ESPE, Saint Pauls, USA) was placed as a temporary filling material. Two weeks later, the patient returned and was asymptomatic. After accessing the tooth and removing the temporary material, GC Fuji 9 glass iomer (GC Corporation, Tokyo, Japan) was placed coronal to the MTA, and the tooth was restored using Clearfil SE bond (Kuraray, Okayama, Japan) bonding agent and Filtek P60 composite resin filling (3M ESPE, St. Paul, USA).

Four months later, the patient returned with swelling and discomfort in the apical area of tooth #20 (Fig. 2b). Radiographic evaluation confirmed an expanding periapical lesion of tooth #20, with slight sensitivity to percussion and grinding but normal probing. Because of a return of symptoms in tooth #20, the lower left area was anesthetized using 2% lidocaine with 1/100,000 epinephrine (Septodont), and the tooth was re-accessed. The canal was disinfected using 1.25% NaOCl. A calcium hydroxide paste (UltraCal XS; Ultradent, South Jordan, USA) was placed as an intracanal medication for 2 weeks to enhance disinfection. The patient returned two weeks later and was asymptomatic. After clot filling, the canal was established, and a collagen matrix plug (CollaCote) was placed, with MTA placed coronally. GC Fuji 9 glass ionomer was placed on top of the MTA plug, and the tooth was restored with Clearfil SE bond and Filtek P60 composite (Fig. 2c, d).

**Figure 3:**
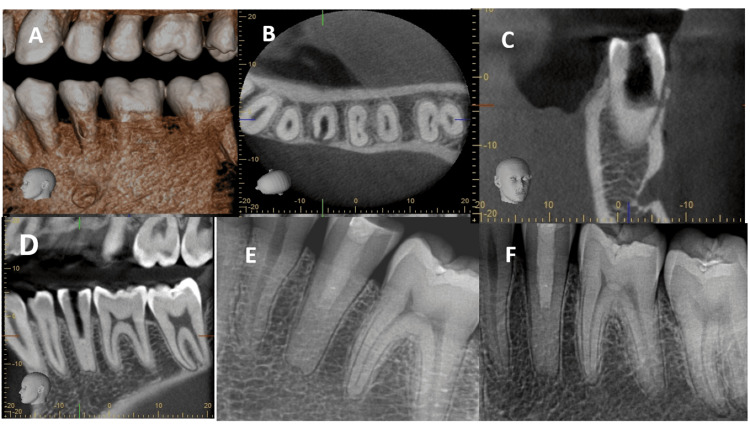
Case 2 - cone-beam computed tomography of tooth #20 (A) Cone-beam computed tomography (CBCT) of tooth #20 (October 2020). (B) Axial view of the same CBCT showing buccal root perforation of tooth #20. (C) Sagittal view of the same CBCT showing buccal root perforation of tooth #20. (D) Coronal view of the same CBCT of tooth #20. (E) Radiograph post-perforation repair using bioceramic material. (F) Recall radiograph (March 2021) showing healing of periapical tissues and no signs of disease progression.

The tooth remained asymptomatic for 2 years (Fig. 2e, f), with continued radiographic monitoring demonstrating increased dentinal wall thickness and root length with near closure of the previous immature apex. Six years after completing the treatment, the patient’s general dentist attempted root canal therapy but was unable to complete the treatment because of canal calcification. The patient was referred again to the practice. Clinical and radiographic evaluation of tooth #20 revealed an empty canal space extending to the mid-root level, with a temporary restoration, sensitivity to percussion and biting, and normal probing (Fig. 2g). Tooth #20 was accessed using the same protocol and anesthesia as in the initial treatment. Upon removal of the temporary restoration, canal bleeding was noted using a dental operating microscope. The procedure was promptly halted, and cone-beam computed tomography was performed, which revealed a buccal perforation (Fig. 3a-3d). The defect was successfully repaired and completely filled with a bioceramic material (Biodentine XP; Septodont, Saint-Maur-des-Fossés, France) after proper disinfection of the root canal with 5.25% NaOCl and 17% EDTA as a final rinse. A final restoration using GC Fuji 9 glass iomer and Filtek P60 composite resin was performed as described earlier (Fig. 3e). At the 6-month recall (Fig. 3f), the patient remained asymptomatic with normal probing. Tooth #20 was fully functional with radiographic evidence of healing and a normal periodontal ligament.

This case demonstrates the long-term success of pulpal regenerative procedures in managing immature non-vital teeth. However, a lack of knowledge of possible late REP outcomes, including heavy root canal calcification, led to improper treatment, resulting in root perforation.

## Discussion

The cases presented herein underscore the critical consequences of limited clinician awareness regarding REPs. Despite favorable long-term outcomes, both REP-treated teeth were subjected to unnecessary and ultimately detrimental retreatment owing to misdiagnosis by practitioners unfamiliar with this biologically based approach.

In both cases described, REP achieved predictable and stable healing over several years; however, subsequent misinterpretation of radiographic findings by clinicians unaware of the normal sequelae of REP led to overtreatment.

Taken together, these observations highlight the need for a simple and reliable method to communicate a patient’s history of regenerative endodontic treatment. The present report aims to address this gap by proposing an RET identification card as a practical tool to support accurate diagnosis and prevent unnecessary retreatment. By providing immediate access to essential procedural information, such a card may help clinicians correctly interpret post‑treatment radiographic findings and preserve the long‑term success of REP‑treated teeth

A key diagnostic challenge following REP lies in differentiating normal healing features from pathological signs. Canal calcification, apical closure without complete root development, and the presence of materials such as MTA or other bioceramic material in the coronal third of the canal may be misinterpreted as indicators of treatment failure or internal resorption. Notably, canal obliteration post-REP often reflects successful hard tissue deposition rather than pathology [5,13-16]. Misunderstanding these features may result in unnecessary retreatment, which carries risks, including root perforation, structural compromise, and failure of biologic repair.

Another major limitation revealed by these cases is the lack of effective long-term communication and documentation. REP is commonly performed in young children, often following traumatic dental injuries. Years later, these patients may not recall the treatment details, and dental records may no longer be accessible. In the absence of clear documentation, future dental professionals may misdiagnose and mistreat these cases. This diagnostic void, compounded by limited undergraduate exposure to REP, places REP-treated teeth at risk. International surveys highlight this educational gap: in the UK, dental students lack foundational training in molecular biology and regenerative techniques [17]; in Saudi Arabia, despite great interest in REP, significant clinical knowledge deficits persist [18]; in India, fewer than half of surveyed students demonstrated adequate understanding, with only a quarter expressing positive perceptions of REP [19].

**Figure 4:**
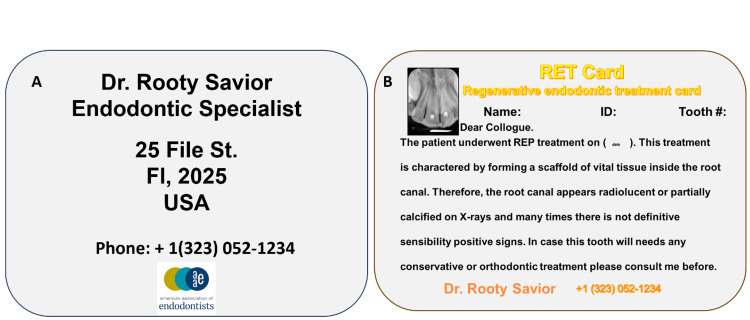
Sample of the suggested regenerative endodontic procedure identification card. (A) Front side of the card. (B) Reverse side of the card. The image of the card has been created by the authors.

To mitigate these risks, we propose the implementation of a “regenerative endodontic treatment identification card” (RET ID card), modeled after medical implant or allergy alert cards (Fig. 4). This card would include essential information such as the procedure performed, materials used, tooth number, and guidance for future care. Such a tool may prevent misdiagnosis and facilitate continuity of care across providers over the lifespan of REP-treated teeth.

Additionally, we recommend adopting a “universal notation system” in dental records (e.g., “REP-2025-MTA+PRF”), improving digital record-keeping, and implementing long-term recall protocols. Broader interdisciplinary awareness is also essential. Orthodontists, prosthodontists, and general dentists involved in subsequent treatment must understand REP’s objectives, expected outcomes, and limitations. Interventions such as aggressive canal preparation, unnecessary retreatment, or post placement can compromise biologically preserved teeth.

In the future, artificial intelligence-based radiographic tools may assist in REP diagnosis by identifying characteristic healing patterns and reducing interpretation errors [20]. Furthermore, current follow-up protocols, typically limited to 12-36 months, may be insufficient. REP-treated teeth can remain functional for decades, yet long-term outcomes and complications remain poorly understood. Extended monitoring, along with patient and parent education regarding the unique nature of REP, is crucial. Patients should be informed that their treated tooth differs from conventionally root canal-treated teeth, and that retreatment should only be considered in the presence of clear pathology or symptoms.

As a case‑based report, the observations presented here have inherent constraints. Case reports cannot establish causality and offer limited generalizability beyond the individual patients described. The absence of a control group restricts comparison with alternative treatment approaches, and the scarcity of similar published cases limits broader contextualization. Although these cases provide valuable insight into diagnostic challenges following REP, conclusions should be interpreted with caution.

## Conclusions

This paper underscores the significant clinical consequences of limited awareness regarding regenerative endodontic procedures (REP), including risks of misdiagnosis, inappropriate retreatment, and the inadvertent reversal of previously successful biologic outcomes. By presenting two illustrative cases, we emphasize the necessity of systematic safeguards such as REP identification cards and the adoption of a universal notation system in dental records (e.g., “REP-2025-MTA+PRF”), which together can strengthen interdisciplinary communication and preserve the integrity of REP-treated teeth.

The implications of this work extend beyond individual case management, highlighting the broader need for standardized documentation and enhanced clinician education. Future research should investigate the efficacy of REP identification tools across diverse practice environments, assess their impact on long-term patient outcomes, and explore strategies for integrating REP awareness into professional training curricula. Such efforts will be essential to ensure that regenerative endodontic procedures are consistently recognized, appropriately managed, and advanced within contemporary dental practice.
